# A Deeper Analysis of Volumetric Relightable Faces

**DOI:** 10.1007/s11263-023-01899-3

**Published:** 2023-10-31

**Authors:** Pramod Rao, B. R. Mallikarjun, Gereon Fox, Tim Weyrich, Bernd Bickel, Hanspeter Pfister, Wojciech Matusik, Fangneng Zhan, Ayush Tewari, Christian Theobalt, Mohamed Elgharib

**Affiliations:** 1 Max Planck Institute for Informatics, Saarland Informatics Campus, Saarbrücken, Germany; 2 Friedrich-Alexander-Universität Erlangen-Nürnberg (FAU), Erlangen, Germany; 3 IST-Austria, Klosterneuburg, Austria; 4 Harvard University, Cambridge, MA USA; 5 MIT CSAIL, Cambridge, MA USA

**Keywords:** Faces, Relighting, Neural radiance fields, Virtual reality

## Abstract

Portrait viewpoint and illumination editing is an important problem with several applications in VR/AR, movies, and photography. Comprehensive knowledge of geometry and illumination is critical for obtaining photorealistic results. Current methods are unable to explicitly model in 3*D* while handling both viewpoint and illumination editing from a single image. In this paper, we propose VoRF, a novel approach that can take even a single portrait image as input and relight human heads under novel illuminations that can be viewed from arbitrary viewpoints. VoRF represents a human head as a continuous volumetric field and learns a prior model of human heads using a coordinate-based MLP with individual latent spaces for identity and illumination. The prior model is learned in an auto-decoder manner over a diverse class of head shapes and appearances, allowing VoRF to generalize to novel test identities from a single input image. Additionally, VoRF has a reflectance MLP that uses the intermediate features of the prior model for rendering One-Light-at-A-Time (OLAT) images under novel views. We synthesize novel illuminations by combining these OLAT images with target environment maps. Qualitative and quantitative evaluations demonstrate the effectiveness of VoRF for relighting and novel view synthesis, even when applied to unseen subjects under uncontrolled illumination. This work is an extension of Rao et al. (VoRF: Volumetric Relightable Faces 2022). We provide extensive evaluation and ablative studies of our model and also provide an application, where any face can be relighted using textual input.

## Introduction

Portrait editing has a wide variety of applications in virtual reality, movies, gaming, photography, teleconferencing, etc. Synthesizing photorealistic novel illuminations and viewpoints of human heads from a monocular image or a few images is still an open challenge.

While there has been a lot of work in photorealistic facial editing (Yamaguchi et al., 2018; Meka et al., 2019; Bi et al., 2021; R et al., 2021b; Pandey et al., 2021; Zhou et al., 2019; Wang et al., 2020; Sun et al., 2019), these methods are usually restricted by sophisticated multi-view input (Meka et al., 2019; Bi et al., 2021; Azinovic et al., 2023; Lattas et al., 2022a), inability to edit the full face region (R et al., 2021b; Yamaguchi et al., 2018; Lattas et al., 2022b; Azinovic et al., 2023; Han et al., 2023; Lattas et al., 2022a) or pure relighting capability without viewpoint editing (Pandey et al., 2021; Wang et al., 2020; Sun et al., 2019; Zhou et al., 2019).

Some recent efforts (R et al., 2021a; Abdal et al., 2021) have shown the ability to edit portrait lighting and viewpoint simultaneously without sophisticated input, while they still suffer from geometric distortion during multi-view synthesis as they rely on a 2D representation.

**Fig. 1:**
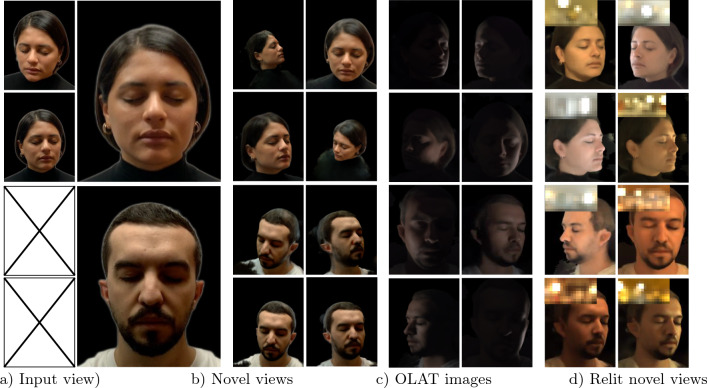
We present VoRF, a learning framework that synthesizes novel views and relighting under any lighting conditions given a single image or a few posed images. VoRF has explicit control over the direction of a point light source and that allows the rendering of a basis of one-light-at-a-time (OLAT) images (**c**). Finally, given an environment map (see **d**, insets) VoRF can relight the input (**d**) by linearly combining the OLAT images

Recently, NeRF (Mildenhall et al., 2020) has proven a powerful 3D representation that is capable of producing novel views at an unprecedented level of photorealism (Mildenhall et al., 2020). NeRF has been applied to tasks like human body synthesis (Su et al., 2021; Liu et al., 2021), scene relighting (Boss et al., 2021; Zhang et al., 2021c; Srinivasan et al., 2021), image compositing (Niemeyer & Geiger, 2021; Yang et al., 2021) and others (Tewari et al., 2022). et al. introduced Neural Light-transport Field (NeLF) (Sun et al., 2021), a NeRF-based approach for facial relighting and viewpoint synthesis that predicts the light-transport field in 3D space and generalizes to unseen identities. However, their method struggles to learn from sparse viewpoints and requires accurate geometry for training. In addition, they need $\ge 5$ views of the input face during testing to avoid strong artifacts.

In this article, we propose a new method that takes a single portrait image as input for synthesizing novel lighting conditions and views. We utilize a NeRF-based volumetric representation and a large-scale multi-view lightstage dataset(Weyrich et al., 2006) to build a space of faces (geometry and appearance) in an auto-decoder fashion using an MLP network, that we call the *Face Prior Network*. This network provides a suitable space to fit any test identity. In addition, our *Reflectance Network* takes a feature vector from the *Face Prior Network* as well as the direction of a point light source as input, to synthesize the corresponding “One-Light-at-A-Time” (OLAT) image. This network is supervised using a lightstage dataset (Weyrich et al., 2006) that captures all aspects of complex lighting effects like self-shadows, diffuse lighting, specularity, sub-surface scattering and higher order inter-reflections. Using OLATs has been shown to improve the quality of relighting (Meka et al., 2019; R et al., 2021b) without assuming a BRDF model or explicit priors. After training, a test identity can be relit by first regressing the corresponding OLAT images for the desired novel viewpoint, which are then linearly combined with any target environment map to synthesize a result (Debevec et al., 2000). In Sect. 3 we show that this principle is indeed compatible with NeRF’s volumetric rendering model (Mildenhall et al., 2020). Our comparisons to previous methods show that our approach produces novel views that are significantly better than those of SOTA methods like PhotoApp (R et al., 2021a). Furthermore, our results are significantly more consistent with the input than those of NeLF (Sun et al., 2021). Our method can operate directly on a monocular image and outperforms NeLF even with 3 input views.

This article extends VoRF (Rao et al., 2022). In particular, we show an application in which any face can be relit using textual input and we provide an extensive study on the impact of design choices, such as the dimentionality of the latent space, the number of training identities, network depth, and the HDR loss function. We are also going to release the code 1 of our implementation.

To summarize, we make the following contributions: (1) We present a NeRF-based approach for full-head relighting that can take a single input image and produces relit results that can be observed from arbitrary viewpoints. (2) We design a dedicated *Reflectance Network* that is built over the *Face Prior Network* that allows our method to learn self-shadows, specularities, sub-surface scattering, and higher order inter-reflections through a lightstage dataset supervision. (3) VoRF is additionally able to synthesize One-Light-at-A-Time 3*D* volume for any given light direction, even though we learn from a dataset that has a limited number of light sources. (4) We demonstrate the use case of relighting any input face using textual input and also provide an exhaustive evaluation of our model.

## Related Work

The literature on portrait editing is vast and here we discuss only methods that are related to relighting. OLAT images generated by a lightstage are popular for capturing the face reflectance details, as pioneered by the seminal work of Debevec et al. (2000). Here, it was shown that such OLAT images can be used as an illumination basis to express an arbitrary environment map through a linear operation. The highly photorealistic relighting achieved by this formulation encouraged further research. This includes methods dedicated for image sequence processing  (Zhang et al., 2021a; Bi et al., 2021), shadow removal (Zhang et al., 2020), capturing high-quality reflectance priorities from monocular images (R et al., 2021b; Yamaguchi et al., 2018) among others (Wang et al., 2020; Meka et al., 2019; Sun et al., 2020; Zhang et al., 2021b; Pandey et al., 2021). Among these, R et al. (2021b) is the closest in problem setting and approach. R et al. (2021b) can regress OLATs for any camera position given a monocular image. But since they rely on the 3DMM model, they can only relight the face interior. The majority of these methods can edit the face interior only (R et al., 2021b; Yamaguchi et al., 2018; Wang et al., 2020) and do not model face exteriors such as hair, VoRF adopts a different strategy. We do not rely on face templates; rather, our approach utilizes the NeRF to learn a 3D radiance field under multi-view image supervision. This method allows us to model the entire head, including the hair. Further, methods (Zhang et al., 2020; Meka et al., 2019; Sun et al., 2020; Zhang et al., 2021b; Pandey et al., 2021; Zhang et al., 2021a) can edit the lighting only while keeping the original camera viewpoint unchanged. The method proposed by Bi et al. (2021) can edit the camera viewpoint and lighting of the full head simultaneously. But, it is person-specific.

Instead of using a lightstage OLAT data, some methods employ illumination models and/or train with synthetic data (Shu et al., 2017; Sengupta et al., 2018; Zhou et al., 2019; Chandran et al., 2022; Lattas et al., 2022b). While these approaches can generalize to unseen identities, they can be limited in terms of photorealism and the overall quality (Shu et al., 2017; Sengupta et al., 2018; Zhou et al., 2019) and some are constrained to editing only the face interior (Lattas et al., 2022b). Recent efforts leverage the generative capabilities of the StyleGAN face model (Karras et al., 2020) to learn from in-the-wild data in a completely self-supervised manner (Tewari et al., 2020; Abdal et al., 2021). More recently, PhotoApp (R et al., 2021a) combined the strength of both lightstage OLAT data and the generative model StyleGAN. Such formulation has two main advantages. First, it achieves strong identity generalization even when training with as few as just 3 identities. Second, it is capable of relighting the full head and editing the camera viewpoint simultaneously. However, as StyleGAN is a 2D generative model, PhotoApp suffers to generate view consistent results in 3D. In contrast, our method learns the prior space in volumetric representation, which generate significantly better view-consistent results. StyleGAN embedding can also change the original identity, leading to unacceptable results. Our method, on the other hand, maintains the integrity of the original identity.

Recently, a multitude of NeRF-based methodologies for general scene relighting have been proposed (Srinivasan et al., 2021; Zhang et al., 2021c; Boss et al., 2021; Martin-Brualla et al., 2021; Rudnev et al., 2022). While NeRV (Srinivasan et al., 2021) necessitates scene illumination as an input, other approaches such as NeRFactor (Zhang et al., 2021c), NeRD (Boss et al., 2021), NeRFW (Martin-Brualla et al., 2021), and NeRF-OSR (Rudnev et al., 2022) can operate with unknown input scene illumination. Notably, the illumination space of NeRFW (Martin-Brualla et al., 2021) is not grounded in physically meaningful semantic parameters. Furthermore, all these aforementioned NeRF-based methods are scene-specific and require multiple images of the scene during the testing phase. In contrast, our approach differs from traditional NeRF by being capable of representing multiple scenes (or subjects), made feasible through the utilization of latent conditioning, as inspired by Park et al. (2019). This advantageous approach provides us the benefits of both NeRF, by relieving us from the necessity of explicit head geometry for face modeling, and latent conditioning, by offering global robustness during the testing phase to manage single image inputs.

Single scene relighting methods such as NeRFW (Martin-Brualla et al., 2021) use latent embeddings to manipulate illumination, while our proposed approach makes use of HDR environment maps. These maps capture real-world illumination, considering each pixel of the environment as a source of light. This results in a lighting environment that is “physically-based”. Further, these environment maps are also “semantically meaningful” because they represent a comprehensible physical reality. The illumination information they provide is grounded in real-world lighting conditions, unlike abstract latent embeddings. This not only makes the maps more intuitively understandable but also ensures that the lighting conditions they provide are relevant and realistic. The closest approach to our problem setting is NeLF (Sun et al., 2021). Based on NeRF, it has a good 3D understanding of the scene. It learns the volume density and light transport for each point in 3D space. NeLF adopts a pixelNeRF-inspired architecture where the density and color values rely heavily on localized image features. As a result, their method struggles to capture global cues and sometimes results in holes in the volume. Their method also requires high-quality geometry for supervision during training and thus fails to learn from sparse viewpoints. It also needs at least 5 viewpoints of the input face during the test otherwise significant artifacts are produced.

Contrary to existing methods, we train a face prior that encodes a joint distribution of identity and illumination, enabling our model, VoRF, to adapt and generalize to unseen subjects and uncontrolled illumination. Generally speaking, human faces follow certain patterns or distributions-for instance, the standard placement of facial features such as two eyes, a nose, and a mouth. As we train the *Face Prior Network* on a variety of subjects, we instill this inductive bias into the model. Given that our scope is restricted to faces, this bias proves to be very beneficial. Additionally, the use of latent codes to represent identity and illumination allows our model to rely on global cues.

This capability permits the synthesis of novel views and relighting effects. Our technique places a strong emphasis on maintaining the integrity of facial geometry during viewpoint interpolation and is capable of relighting the entire head.

A notable feature is its ability to operate using as few as a single monocular image during testing. Additionally, our method presents innovative latent interpolation capabilities, which allow for the rendering of unseen identities and illumination conditions during the testing phase.

## Face Reflectance Fields

Obtaining complex lighting conditions by linearly combining OLAT images according to environment maps is a principle that is well-studied in the literature (Debevec et al., 2000). In this section, we show that this principle is actually compatible with NeRF’s volumetric rendering model (Mildenhall et al., 2020).

Debevec et al. (2000) argue that under the assumption that all sources of incident light are sufficiently far away from the face, we can describe lighting conditions by a function $L_\text {inc}(\omega )$, that only depends on a direction $\omega \in S$ from which radiance is incident and maps this direction to the total amount radiance reaching the face from that direction. $S$ is the set of all directions of incoming radiance.

We introduce a combination of a *volume density function* (Mildenhall et al., 2020) and a *reflectance field* (Debevec et al., 2000), that we call *volumetric reflectance field*: A volumetric reflectance field is a pair $(\sigma , R)$, where the *volume density function* $\sigma : \mathbb {R}^ 3 \rightarrow \mathbb {R}$ maps scene points to density values and the function $R(\omega , \textbf{x}, \textbf{d})$ indicates the fraction of $L_\text {inc}(\omega )$ that is reflected from point $\textbf{x}$ in the direction $\textbf{d}$.

The additive property of light transport allows us to describe the total amount $L_\text {out}(\textbf{x}, \textbf{d})$ of radiance reflected out of point $\textbf{x}$ in the direction $\textbf{d}$ as

1 $$\begin{aligned} L_\text {out}(\textbf{x}, \textbf{d}):= \int \limits _{\omega \in S} R(\omega , \textbf{x}, \textbf{d}) \cdot L_\text {inc}(\omega ) \; d\omega \end{aligned}$$

We assume that image formation follows a perceptive camera model, as described by Mildenhall et al. (2020), i.e. we assume a ray $r_{\textbf{o}, \textbf{d}}(t) = \textbf{o}+ t \textbf{d}$ being shot through a camera pixel into the scene, and describe the amount of radiance accumulated along this ray as

2 $$\begin{aligned} \begin{aligned} L(r)&:= \int \limits _{t_\text {n}}^{t_\text {f}} T(t) \cdot \sigma (r(t)) \cdot L_\text {out}(r(t), d)\; dt \; \\ \text {with} \; T(t)&:= \exp \left( - \int \limits _{t_\text {n}}^{t} \sigma (r(s)) ds\right) \end{aligned} \end{aligned}$$

where $t_\text {n}, t_\text {f}$ are the bounds within which the entire face is contained.

In order to bridge the gap between the OLAT conditions of the dataset and real-world lighting conditions, we discretize the dense set of incident light directions $S$ to a finite set $I$, with one direction $i\in I$ per OLAT light source where $S_i\subseteq S$ represents a subset. We now approximate the following:

3 $$\begin{aligned} L_\text {out}(\textbf{x}, \textbf{d}) \approx \sum _{i\in I} R(\omega _i, \textbf{x}, \textbf{d}) \cdot L_\text {inc}(i) \end{aligned}$$

where $\omega _i$ is the incident light direction of OLAT light source *i* and $L_\text {inc}(i):= \int _{\omega \in S_i} L_\text {inc}(\omega )$ is the discretized version of $L_\text {inc}$.

The property of OLATs that allow to compose complex lighting conditions can now be derived as follows:

*Under OLAT Conditions*, i.e. when the face is illuminated from only one single light source, there exists a single $i\in I$ that contributes some radiance $\mathbb {L}_i:= L_\text {inc}(i)$ (i.e. only lamp $i$ is turned on), while for all $j\ne i$ we have $L_\text {inc}(j) = 0$. Thus, for a given ray $r$ with origin $\textbf{o}$ and direction $\textbf{d}$, the accumulated radiance $L(r)$ is approximated by

4 $$\begin{aligned} L(i, r):= \int \limits _{t_\text {n}}^{t_\text {f}} T(t) \cdot \sigma (r(t)) \cdot R(\omega _i, r(t), \textbf{d}) \cdot \mathbb {L}_i\; dt \end{aligned}$$

*Under Non-OLAT Conditions*, all we know is that $\forall i\in I$ there must exist some factor $f_i$, s.t. $L_\text {inc}(i) = f_i\cdot \mathbb {L}_i$. With the abbreviation $a(t):= T(t) \cdot \sigma (r(t))$ we can thus equate

5 $$\begin{aligned} \begin{aligned} L(r)&\approx \int \limits _{t_\text {n}}^{t_\text {f}} a(t) \cdot \sum _{i\in I} R(\omega _i, r(t), \textbf{d}) \cdot f_i\cdot \mathbb {L}_i\; dt \\&= \sum _{i\in I} f_i\cdot \int \limits _{t_\text {n}}^{t_\text {f}} a(t) \cdot R(\omega _i, r(t), \textbf{d}) \cdot \mathbb {L}_i\; dt \\ {}&= \sum _{i\in I} f_i\cdot L(i, r), \end{aligned} \end{aligned}$$

where $L(i, r)$ is the amount of radiance that, originating from light source *i*, emerges from the scene along ray $r$.

Equation 5 shows that under the stated assumptions we can render the face under any given lighting specification $(f_i)_{i\in I}$ just as a linear combination of OLAT images. The errors caused by the approximations ($\approx$) in the derivations above reduce as we increase the number of OLAT directions that are used to discretize $S$. Similar equations are known in the literature (Debevec et al., 2000), showing that under the stated assumptions we can render the face under any given lighting specification $(f_i)_{i\in I}$ just as a linear combination of OLAT images.

**Fig. 2:**
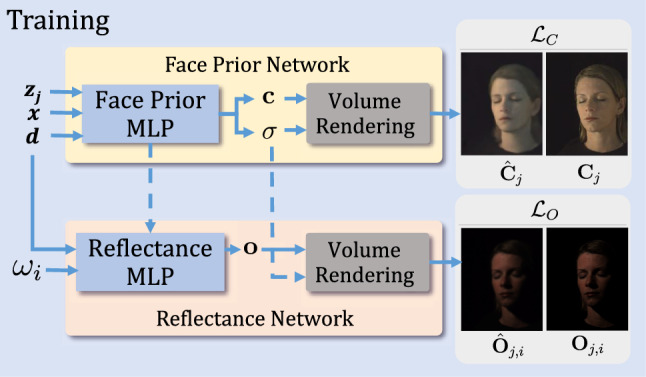
Our *Face Prior Network* learns to decode latent codes $\textbf{z}_j$ to estimate radiance and volume density for each point in 3D space. Our *Reflectance Network* learns to synthesize OLAT images of the face (Color figure online)

Our NeRF-based (Mildenhall et al., 2020) model in Sect. 4 learns functions of the form $F_\Theta (\textbf{x}, \textbf{d}) = (L_\text {out}(\textbf{x}, \textbf{d}), \sigma (\textbf{x}))$, based on latent codes for the facial identity and lighting conditions, making Eq. 2 computationally tractable. To train our face prior network (see Sect. 4) and to evaluate our method, we use HDR environment maps from the Laval Outdoor dataset (Hold-Geoffroy et al., 2019) and the Laval Indoor HDR dataset (Gardner et al., 2017) to obtain coefficients $f_i$. This allows us to turn the OLAT basis images into depictions of faces under real-world lighting conditions and we generate 600 relit images for each subject. Refer to Sect. 7.4 for more details.

## Method

We address the problem of simultaneous portrait view synthesis and relighting. Given a small set of $N\ge 1$ input images along with their camera parameters, we build a *Face Prior Network*($\mathcal {P}$) and a *Reflectance Network*($\mathcal {R}$) utilizing NeRF-based representation. Firstly, the $\mathcal {P}$ is modeled in an auto-decoder fashion to learn a prior over human heads under various illumination conditions and this formulation allows VoRF to generalize to novel test identities. Furthermore, to model face reflectance that can re-illuminate a face for several viewpoints, we design a $\mathcal {R}$ that learns to predict OLAT images. Using Eq. 5, we linearly combine these OLAT images with HDR environment maps to render novel views of a given face, under new lighting conditions. An overview of our method can be found in Fig. 2.

### Learning Face Priors

Neural Radiance Fields (Mildenhall et al., 2020) learns a coordinate-based representation of each scene by mapping 3D coordinates $\textbf{x}\in \mathbb {R}^3$ and direction $\textbf{d}\in {\mathcal {S}}^2$ to the densities and radiance values. However, NeRF by design is able to optimize a single scene at a time. To combat this and obtain a distribution over the entire space of faces and illumination conditions, we use an auto-decoder formulation. More specifically, we first prepare a dataset by combining a set of environment maps with OLAT images acquired from lightstage resulting in $\mathbb {J}$ combinations. For each combination $j\in \mathbb {J}$, we obtain image $\textbf{C}_j$ and a corresponding latent code $\textbf{z}_j$. The latent code $\textbf{z}_j$ is partitioned into identity and illumination components as $\textbf{z}^ \text {id}_j$ and $\textbf{z}^ \text {env}_j$ respectively. We initialize the latent codes from a multivariate normal distribution and observe that separating the components individually leads to faster convergence during the training process (see Sect. 7.4). We design the *Face Prior Network* to take the latent code $\textbf{z}_j$ along with $\textbf{x}$, $\textbf{d}$ as inputs and predict radiance $\textbf{c}$ as well as volume density $\sigma$ for every point in 3*D* space. We represent the *Face Prior Network* as $\mathcal {P}_{\Theta _\mathcal {P}}(\textbf{z}_j, \textbf{x}, \textbf{d}) = (\textbf{c}, \sigma )$. Following NeRF, the network weights $\Theta _\mathcal {P}$ along with the latent codes $\textbf{z}$ are optimized jointly to regress the color values with a mean squared objective function as follows:

6 $$\begin{aligned} \mathcal {L}_\text {C}:= \sum \limits _{j\in \mathbb {J}}\Vert \hat{\textbf{C}}_j- \textbf{C}_j \Vert _2^2 \end{aligned}$$

where $\hat{\textbf{C}}_j$ is the image obtained by volume rendering based on $\mathcal {P}_{\Theta _\mathcal {P}}(\textbf{z}_j, \;.\;, \;.\;)$.

Drawing inspiration from (Park et al., 2019), we initialize the latent codes to be derived from a zero-mean multivariate Gaussian. This prior enforces that identity and illumination codes should reside within a compact manifold. Such a notion ensures that latent codes are concentrated, leading to smooth interpolation and convergence to an optimal solution. We maintain this by implementing an L2 regularization Eq. 7 to prevent the distribution from growing arbitrarily large. Based on our empirical results, this simple constraint proved to be sufficient in learning a useful latent distribution.

7 $$\begin{aligned} \mathcal {L}_\text {reg}= \sum \limits _{j\in \mathbb {J}} \Vert \textbf{z}^ \text {id}_j \Vert _2^2 + \Vert \textbf{z}^ \text {env}_j \Vert _2^2 \end{aligned}$$

### Synthesizing New OLAT Images

To model a reflectance field of the faces, we propose a *Reflectance Network*($\mathcal {R}$) that learns a volumetric reflectance field by utilizing the $\sigma$ predictions provided by $\mathcal {P}$ (see Sect. 3). For an OLAT light source $i$, we consider the incident light direction $\omega _i$ as an input to the $\mathcal {R}$. To synthesize OLAT images, we design the $\mathcal {R}$ based on NeRF and directly regress the radiance values $\textbf{o}$.

$\mathcal {R}$ models face reflectance by taking into account the incoming light direction and features derived from the $\mathcal {P}$. As the $\omega _i$ is already known, the network needs information related to face geometry to model the reflectance function and hence the outgoing light. By design, we predict density from the output of the 9th layer of the $\mathcal {P}$. Hence to ensure reliable geometry information is passed on to the $\mathcal {R}$ we extract features from this layer.

We also provide the viewing direction $\textbf{d}$ as input to capture view-dependent effects. Thus, *Reflectance Network* learns a function $\mathcal {R}_{\Theta _\mathcal {R}}$, parameterized by $\Theta _\mathcal {R}$ and is given as follows: $\mathcal {R}_{\Theta _\mathcal {R}}(\omega _i, \mathcal {F}_\mathcal {P}(\textbf{z}_j, \textbf{x}, \textbf{d}), \textbf{d}) = \textbf{o}$. To synthesize an OLAT image $\hat{\textbf{O}}_{j, i}$ along the light direction $i$ for $j\in \mathbb {J}$, we combine $\textbf{o}$ with the volume density $\sigma$ predicted from $\mathcal {P}$. The dotted line in Fig. 2, connecting density ($\sigma$) from the $\mathcal {P}$ to the volume rendering block of the $\mathcal {R}$, demonstrates this connection.

This design choice can be intuitively understood. Regardless of the specific OLAT lighting condition, the subject, and therefore, the face geometry, remains constant. We enforce this fixed geometry by ensuring the $\mathcal {R}$ uses the density information from the previous stage. We’ve found in our work that this approach facilitates faster learning. This is because it allows the $\mathcal {R}$ to differentiate between shadow and geometry within darker regions of the OLAT images thereby avoiding shape-illumination ambiguity. $\Theta _\mathcal {R}$ is optimized by minimizing HDR-based loss inspired by Mildenhall et al. (2022) and $S$ is a stop gradient function:

8 $$\begin{aligned} \mathcal {L}_\text {O}:= \sum \limits _{j\in \mathbb {J}} \left\| \frac{\hat{\textbf{O}}_{j, i} - \textbf{O}_{j, i}}{S(\hat{\textbf{O}}_{j, i}) + \epsilon } \right\| _2^2 \end{aligned}$$

where $\textbf{O}_{j, i}$ is the ground truth OLAT image from the dataset that is used in the construction of $\textbf{C}_j$. This loss function is especially suited for handling the semi-dark lighting conditions of OLAT images. Our HDR lightstage dataset predominantly consists of dark regions and utilizing an L2 loss function results in muddy artifacts in those regions (Mildenhall et al., 2022). In contrast, the HDR-Loss divides the absolute error by the brightness of the ground truth image giving a higher weight value for darker regions. Thus, utilizing this loss function helps to recover high contrast differences in dark regions.

### Training

NeRF-based methods typically require dense camera views of the scene to faithfully represent the scene without cloudy artifacts. As our dataset has a limited number of views, we make use of hard-loss (Rebain et al., 2022) to avoid cloudy artifacts. We consider, as in previous work, the *accumulation weights* $w_{r, k}$ that are computed during volume rendering, for a given ray *r* (see (Rebain et al., 2022)). Imposing ${\mathbb {P}}(w_{r, k}) \propto e^{- |w_{r, k} |} + e^{- |1 - w_{r, k} |}$ for the probabilities of these weights, we minimize

9 $$\begin{aligned} \mathcal {L}_\text {h}= \sum \limits _{r, k}- \log ( {\mathbb {P}}(w_{r, k})) \end{aligned}$$

which encourages the density functions implemented by $\mathcal {P}$ to produce hard transitions. We apply this loss during the synthesis of both $\hat{\textbf{C}}_j$ and $\hat{\textbf{O}}_{j, i}$, which helps to avoid cloud artifacts surrounding the face.

*Training Scheme* After the initial stage of training that ensures a reasonable level of convergence of $\mathcal {P}$, we proceed to jointly optimize both the $\mathcal {P}$ and the $\mathcal {R}$. Our overall training loss function now is $\mathcal {L}= \alpha \mathcal {L}_\text {C}+ \beta \mathcal {L}_\text {O}+ \gamma \mathcal {L}_\text {reg}+ \delta \mathcal {L}_\text {h}$ with hyper weights $\alpha , \beta , \gamma , \delta$.

It’s noteworthy from our experiments that we didn’t need to adjust the hyperparameters $\alpha , \gamma$, and $\delta$ during this phase of joint training. They remained consistent, indicating the robustness of our model and training process.

### Test

Following the training phase, where our proposed model learns from 302 subjects, each captured under 600 random natural illuminations, the model learns to differentiate between identity and illumination effectively. This distinction is robust enough to generalize to a test subject. During the test-time optimization, the $\mathcal {P}$ assists in distilling identity-specific and illumination-specific details into $\varvec{\textbf{z}}_{id}$ and $\varvec{\textbf{z}}_{env}$ respectively.

Having trained the networks on a large-scale dataset, we operate under the assumption that the test subject’s identity and illumination are close to the training distribution. Therefore, the features extracted from the $\mathcal {P}$ facilitate the $\mathcal {R}$ in modeling the reflectance of the test subject and predicting One-Light-at-A-Time (OLAT) images. It is important to note that our $\mathcal {R}$ does not directly depend on $\varvec{\textbf{z}}_{env}$ to model face reflectance. Instead, it primarily relies on identity-specific geometric details, encoded in $\varvec{\textbf{z}}_{id}$, to model the reflectance function.

**Fig. 3:**
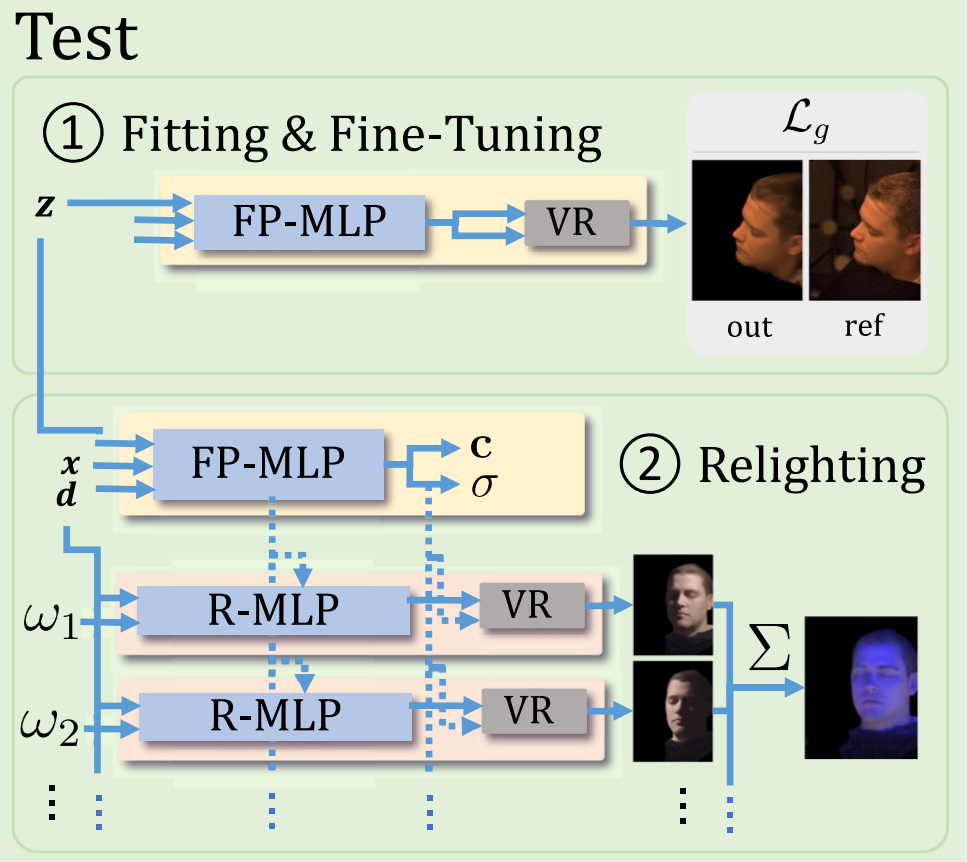
To reconstruct an unseen test face, we optimize latent code $\textbf{z}$ and fine-tune the *Face Prior Network*, We can relight the reconstructed face by having the *Reflectance Network* produce a basis of OLAT images (step 2), that we linearly combine into any desired lighting condition. In this figure, MLP’s with the same label: “R-MLP” share their weights

Given a small set of $N\ge 1$ input images of an unseen identity under unseen lighting conditions, we fit $\textbf{z}$ and fine-tune $\Theta _\mathcal {P}$ by minimizing (using backpropagation)

10 $$\begin{aligned} \mathcal {L}_\text {g}:= \alpha \mathcal {L}_\text {C}+ \gamma \mathcal {L}_\text {reg}+ \delta \mathcal {L}_\text {h}\end{aligned}$$

where the input images now take the place of the $\textbf{C}$ that were used during training. Note, that first, we update only $\textbf{z}$ for 10,000 iterations (learning rate $1 \times 10^{-3}$), to make sure that it lies well within the learned prior distribution. Then, assuming that the fitting step has converged, continue to jointly update $\textbf{z}$ and $\Theta _\mathcal {P}$ for 3,000 iterations (learning rate $3 \times 10^{-6}$). We demonstrate the significance of this two-step approach as an ablation study in the Sect. 7.1.

With $\textbf{z}$ and $\Theta _\mathcal {P}$ optimized in this way (part $\textcircled {1}$ in Fig. 3), we can already render the face under novel views. In order to be able to also change lighting (part $\textcircled {2}$ in Fig. 3), we use $\mathcal {R}$ to render an OLAT basis that by Eq. (5) we can use to synthesize any given lighting conditions.

## Lightstage Dataset

**Fig. 4:**
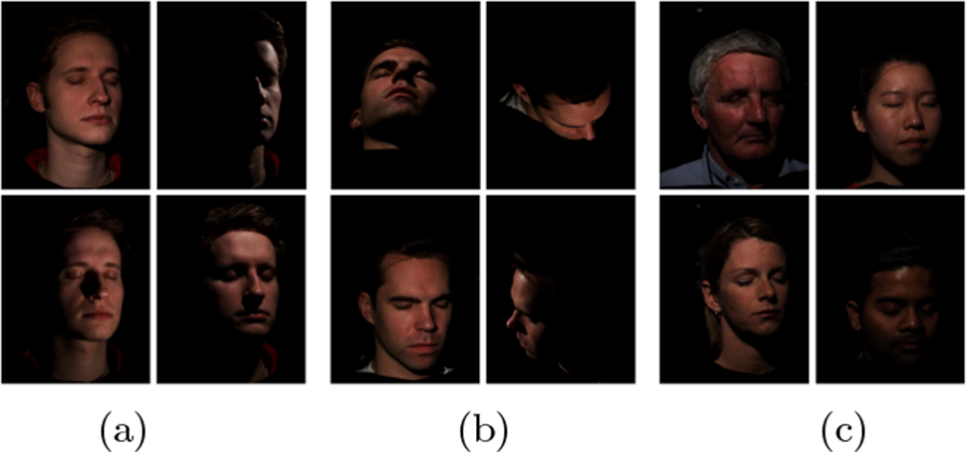
We use a light stage dataset (Weyrich et al., 2006) that provides 150 different lighting conditions (**a**), 16 camera angles (**b**), and 353 subjects (**c**). We brightened the images here, for better visualization

**Fig. 5:**
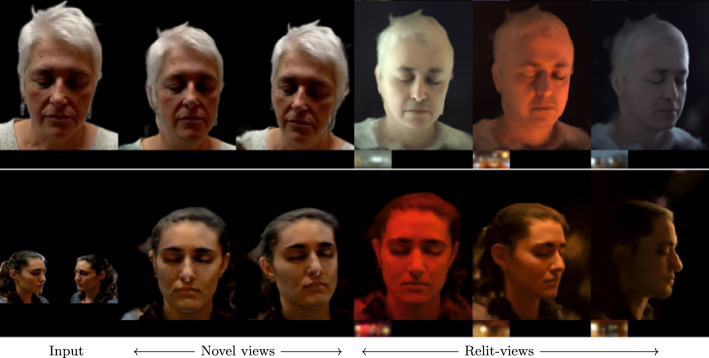
Novel view synthesis + relighting on unseen identities from the H3DS (Ramon et al., 2021) dataset. We show results obtained by using a single image (top) and two images (bottom). Target environment maps are shown in the insets. Our technique performs photorealistic novel view-synthesis and relighting

We utilize a lightstage dataset (Weyrich et al., 2006) of 353 identities, illuminated by 150 point light sources and captured by 16 cameras. The light sources are distributed uniformly on a sphere centered around the face of the subject. For every subject, each camera captures 150 images (1 per light source). All the images are captured with the subject showing a neutral expression with their eyes closed. While capturing each frame, the light sources were turned on one at a time, thus generating one-light-at-a-time (OLAT) images. Figure 4 gives an impression of the dataset.

### Lightstage Test Dataset

For experiments that require a ground-truth reference, we created such a reference by combining lightstage images according to different environment maps: We randomly sampled 10 unseen identities from the lightstage dataset and synthesized naturally lit images using 10 randomly chosen unseen HDR environment maps, from the Laval Outdoor dataset (Hold-Geoffroy et al., 2019) and the Laval Indoor HDR dataset (Gardner et al., 2017). For all quantitative and qualitative experiments, we evaluate only the held-out views. For instance, given that the lightstage dataset has a total of 16 camera viewpoints, an evaluation method that takes three input views would be evaluated on the remaining 13 held-out views.

## Results

We evaluate our method qualitatively and quantitatively to demonstrate the efficacy of our method using our lightstage dataset, see Sect. 6.1. Additionally, we qualitatively evaluate our method on H3DS (Ramon et al., 2021), a naturally lit multi-view dataset.

To the best of our knowledge, NeLF and VoRF were among the first 3D methodologies capable of generalizable, simultaneous viewpoint and illumination editing for full human heads using just images. Moreover, IBRNet (Wang et al., 2021) is a recognized state-of-the-art method for view synthesis that can generalize across multiple scenes, making it a relevant comparison point. Following NeLF we use a combination of IBRNet and SIPR (Sun et al., 2019) for simultaneous view synthesis and relighting. Finally, PhotoApp (R et al., 2021a) utilizes the 2D StyleGAN latent space (Karras et al., 2020) and learns to edit the illumination and camera viewpoint in this space. In summary, we compare against three state-of-the-art methods: (1) NeLF (2) IBRNet + SIPR, and (3) PhotoApp.

To accurately evaluate the effectiveness of our proposed approach, it is critical to compare it with the state-of-the-art methods using the same dataset for both quantitative and qualitative assessments. Hence, for a fair comparison, we retrain NeLF, IBRNet, SIPR, and PhotoApp with our lightstage dataset. All the methods are retrained as suggested in the original works. Further, we discussed with the authors of NeLF and PhotoApp to validate our findings and ensure the correctness of our reimplementation. The authors corroborated our findings, confirming their consistency and accuracy. In light of the lack of existing open-source multi-view lightstage datasets and global latent code-based generalizable NeRFs, we maintain that the comparison is fair and appropriate.

Finally, we perform ablation studies on the various design choices of our framework and discuss their significance in the Sects. 7 and 8.

**Table 1:** Comparing against NeLF (Sun et al., 2021) (requires at least 5 input views), IBRNet (Wang et al., 2021) and SIPR (Sun et al., 2019) in view synthesis and relighting

	View synthesis						View synthesis and relighting					
	NeLF		IBRNet		Ours		NeLF		IBRNet+SIPR		Ours	
Input	PSNR	SSIM	PSNR	SSIM	PSNR	SSIM	PSNR	SSIM	PSNR	SSIM	PSNR	SSIM
5-views	22.01	0.80	24.38	0.82	**27**.**45**	**0**.**84**	21.34	0.79	19.63	0.75	**24**.**16**	**0**.**81**
3-views	20.57	0.75	22.0	0.76	**26**.**67**	**0**.**82**	19.72	0.75	18.38	0.73	**22**.**80**	**0**.**76**
2-views	19.63	0.70	20.34	0.71	**25**.**44**	**0**.**79**	19.06	0.69	17.01	0.71	**22**.**15**	**0**.**74**
1-view	N/A	N/A	N/A	N/A	**22**.**49**	**0**.**77**	N/A	N/A	N/A	N/A	**20**.**21**	**0**.**69**

Our technique outperforms related methods regardless of the number of input views (see bold)

### View Synthesis and Relighting

In this section we present the results for view synthesis and relighting to demonstrate that our method can synthesize novel lighting conditions of the subject at novel viewpoints.

**Fig. 6:**
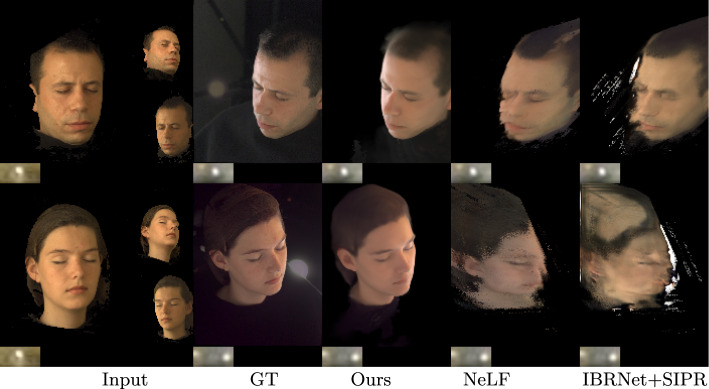
A sample result on the lightstage test set, with ground truth. Our technique produces novel view synthesis and relighting that clearly outperform NeLF (Sun et al., 2021) and IBRNet (Wang et al., 2021) + SIPR (Sun et al., 2019)

Figure 5 shows novel view synthesis and relighting produced by our technique. Here, we present results with single input view (top) and two input views (bottom). We observe that our method produces photorealistic renderings that are view-consistent. Our method maintains the integrity of the input identity and recovers the full head, including hair. It also maintains the integrity of the facial geometry while relighting at extreme views (third and fourth row, last column in Fig. 5).

**Fig. 7:**
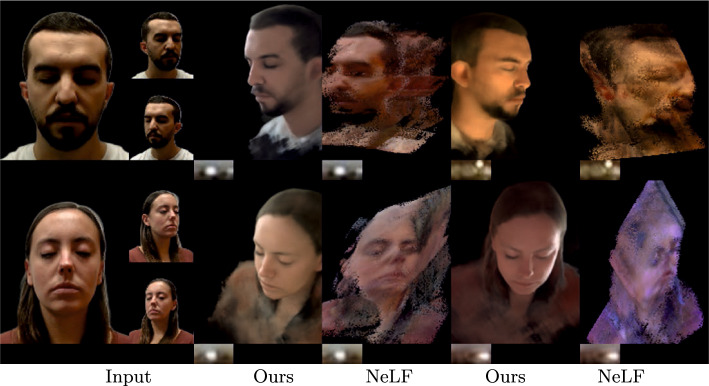
Comparison of our method and NeLF (Sun et al., 2021) on the H3DS (Ramon et al., 2021) dataset for simultaneous novel view synthesis and relighting. Our technique outperforms NeLF in terms of relighting quality, especially at views that are far from the training set

Our *Reflectance Network* has the ability to synthesize subjects corresponding to arbitrary light directions and enable us to relight them using any HDR environment maps following Eq. 5. To achieve this, our technique predicts the 150 OLAT images as the light basis of the lightstage. In Sect. 2 we show that through our rendered OLATs we are able to reproduce view-dependent effects, specular highlights and shadows.

### Comparison to Related Methods

We quantitatively and qualitatively compare against the state-of-the-art view synthesis and relighting methods. All the quantitative evaluations are on the lightstage test set as detailed in  Sect. 5.1. We summarize our quantitative evaluations in Table 1 in terms of average PSNR and SSIM over all the test images.

First, we compare our method for the view-synthesis task with a different number of input views. Next, with the same test setup we evaluate for the task of simultaneous view synthesis and relighting. For both tasks, we observe that our method convincingly outperforms NeLF, IBRNet, and IBRNet $+$ SIPR.

We posit that the limitations of other methods, such as NeLF and IBRNet, are not due to the nature of the training dataset itself but rather due to their design. Both NeLF and IBRNet are reliant on local features for reasoning about geometry, which demands 3–5 images with viewpoints not too far apart during evaluation. In contrast, our approach relies on global features and can operate effectively with a single input view.

**Fig. 8:**
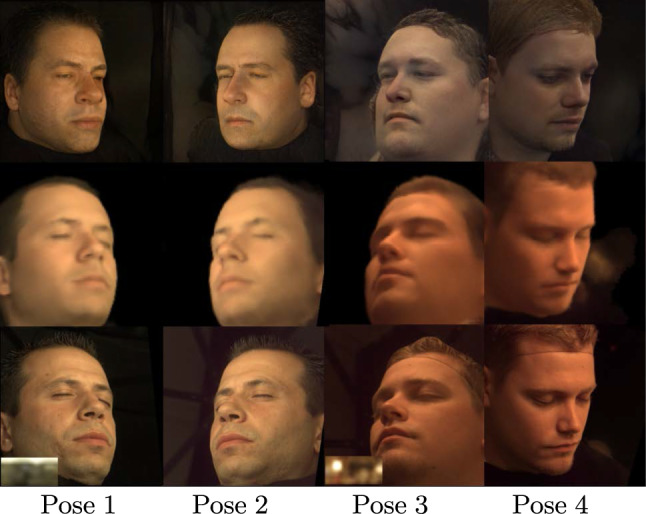
Comparison of PhotoApp (R et al., 2021a) (top row) and our (middle row) method for simultaneous view synthesis and relighting on the lightstage test set with single view input. PhotoApp suffers from strong identity alternations, pose inaccuracies, and view-inconsistent lighting. In contrast, our method produces more view-consistent and visually pleasing results, closer to the ground truth (bottom row)

As a direct consequence, neither NeLF nor IBRNet can handle single-input images which limits their application to multi-view setups. High evaluation scores indicate that our method recovers decent geometry and synthesizes better-quality relighting. These results can be more easily understood in Fig. 6, where we clearly observe our renderings match the ground truth more closely than the baseline methods. While IBRNet and NeLF have different design principles relative to VoRF, our comparison is intended to highlight the inherent design limitations of the methods, which rely on local image features for geometry inference and thus are significantly dependent on dense multi-view inputs during testing for unseen subjects. We argue that these limitations are inherent in any method that employs local-image-aligned CNN features to learn a NeRF representation and are not a failure due to the nature of the training dataset. In fact, our reimplementations all the baselines show convergence during training with our lightstage dataset. Additionally, In light of the lack of existing global latent code-based NeRF methods that can generalize to new scenes, we chose IBRNet as an additional benchmark for our evaluations. The aim is not to discredit other methods but to provide a more holistic understanding of the trade-offs involved in different approaches to the challenging problem of simultaneous viewpoint and illumination editing.

We additionally compare against NeLF on H3DS dataset (see Fig. 7) where our approach clearly performs better. We argue this is due to NeLF’s inability to recover decent geometry from sparse views. Likewise, IBRNet fails to construct multi-view consistent geometry under sparse views. Further with IBRNet+SIPR, we observe that SIPR depends on the viewpoint, which breaks down the multi-view consistent relighting. Finally, we compare against PhotoApp in Fig. 8. PhotoApp inherits the limitations of the StyleGAN space, specifically, the inversion step which modifies the input identity. Such modifications lead to highly inconsistent results limiting the application of PhotoApp. In contrast, our approach produces view-consistent results that resemble ground truth.

## Ablations

Our results in Sect. 6 demonstrate that our method outperforms existing state-of-the-art approaches. In this section, we further evaluate the design choices.

**Table 2:** Omitting the fine-tuning stage of our optimization process at test time (see Sect. 7.1) leads to significantly lower scores (“Fit Only”)

	View synthesis			View synthesis + Relighting		
	Fit Only	Single *z*	Full Model	w/o $\mathcal {L}_\text {O}$	w/o $\mathcal {R}$	Full model
PSNR	22.39	24.53	**26**.**67**	20.71	20.81	**22**.**80**
SSIM	0.71	0.82	**0**.**82**	0.61	0.72	**0**.**76**

We evaluate the two latent space design choices (“Single *z*”). We observe that using a disentangled latent space design (see Sect. 7.4) leads to improved performance, mainly attributed to a better face prior representation that helps in generalization. Our evaluations show that using $\mathcal {L}_\text {O}$ instead of MSE loss (“w/o $\mathcal {L}_\text {O}$”) to supervise HDR improves the performance of our method (see bold). We quantitatively demonstrate the significance *Reflectance Network* (“w/o $\mathcal {R}$”). Clearly having a dedicated *Reflectance Network* improves the relighting quality

### Significance of Two-Stage Optimization

We investigate the efficacy of our two-stage optimization process in reconstructing a novel test identity for the task of novel view synthesis. At test time, our optimization process consists of two stages: *fitting* the latent code $\textbf{z}_{test}$ to the test subject and a subsequent *fine-tuning* process where we jointly optimize $\textbf{z}_{test}$ and the weights of network $\mathcal {P}$, i.e., $\Theta _\mathcal {P}$, to refine the reconstruction. We perform the *fitting* process for 10,000 iterations with a learning rate of $1\times 10^{-3}$ to ensure that $\textbf{z}_{test}$ lies in the learned face prior distribution. After achieving convergence, we reduce the learning rate to $1\times 10^{-6}$ and jointly optimize $\textbf{z}_{test}$ and $\Theta _\mathcal {P}$ for 3000 iterations. We do not modify the weights of $\mathcal {R}$ in either stage of optimization.

**Fig. 9:**
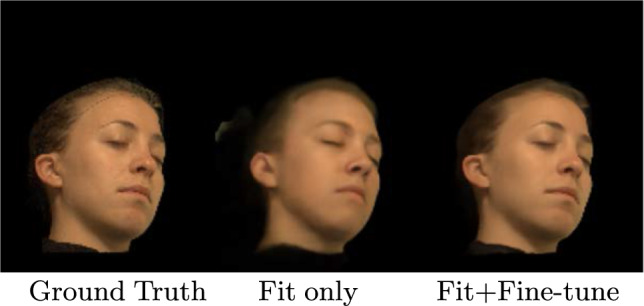
Performing the two-step optimization improves the overall quality by recovering identity-specific high-frequency details. We show results from a novel viewpoint

**Fig. 10:**
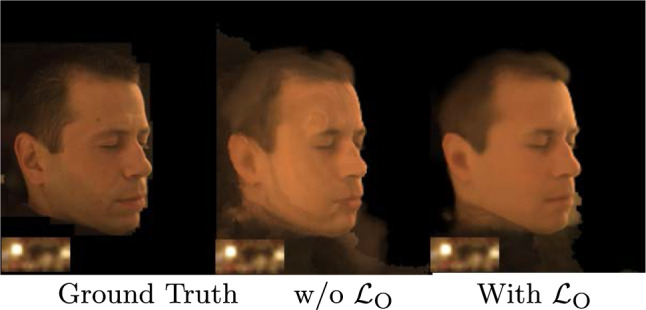
Impact of $\mathcal {L}_\text {O}$. We observe that without $\mathcal {L}_\text {O}$ the relighting quality is poorer due to deterioration in the OLATs predicted by *Reflectance Network*

To assess the impact of this design choice on novel view synthesis, we compare the performance of *Full Model (Fit + FineTune)* to that of *Fit only* on our lightstage test dataset, as shown in Table 2. Our results demonstrate that the two-stage optimization process leads to superior performance. Specifically, in Fig. 9, we observe that the fitting stage recovers an approximate face geometry, while the fine-tuning stage restores identity-specific fine details to the reconstruction.

In conclusion, our results demonstrate that the two-stage optimization process yields improved performance, outperforming the *Fit only* baseline on our lightstage test dataset.

### Significance of $\mathcal {L}_\text {O}$

NeRF in the dark (Mildenhall et al., 2022) proposes a modified MSE loss function (Sect. 4.2) that is better suited for training in HDR space. We utilize this loss function (as denoted by $\mathcal {L}_\text {O}$) for HDR OLAT supervision during training of our *Reflectance Network*. Table 2 indicates that the use of a naive MSE loss instead of $\mathcal {L}_\text {O}$ results in poorer relighting quality. This is attributed to the deterioration in OLAT quality as MSE is not suitable for supervision in HDR space.

### Significance of the *Reflectance Network*

We investigate the significance of the *Reflectance Network* in our proposed framework for the task of simultaneous view synthesis and relighting portraits.  In this ablation study, we compare the performance of using only $\mathcal {P}$ with our proposed framework involving both $\mathcal {P}$ and $\mathcal {R}$. We initialize $\varvec{\textbf{z}}_{env}$ from an environment map, while $\varvec{\textbf{z}}_{id}$ is initialized from a latent embedding, following the method of our original design. Despite the difference in initialization, the optimization process applied to these latent vectors remains the same when fitting the model to an unseen subject.

**Fig. 11:**
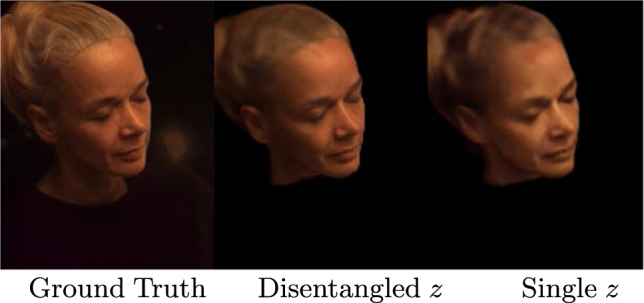
We compare the design choice *Disentangled Latent code* (i.e. separate latent codes for identity and illumination) to the alternative *Single Latent code* (i.e. one latent code *per combination* of identity and illumination), by evaluating for the task of view synthesis on our lightstage dataset. The disentangled version leads to better reconstructions

**Fig. 12:**
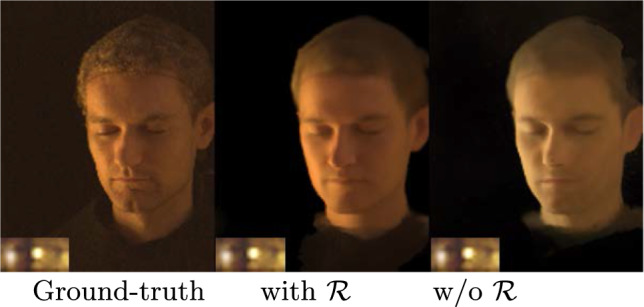
Left: Performing the two-step optimization improves the overall quality during view-synthesis. Right: Removing the *Reflectance Network* (“w/o $\mathcal {R}$”) leads to a clear loss in quality during relighting

By directly feeding the environment map into the model, we hypothesize that the network learns to parse and encode scene illumination from $\varvec{\textbf{z}}_{env}$ directly, while identity-specific information is learned through the optimization process. During the training of the $\mathcal {P}$, we expose each subject to 600 different illuminations, some of which are repeated across multiple subjects, allowing the network to learn the disentanglement of identity and illumination.

To perform viewpoint editing and relighting using only $\mathcal {P}$, we modify the network architecture slightly. Instead of using the illumination latent $\textbf{z}_\text {env}$, we directly input the downsampled HDR environment map and train $\mathcal {P}$. This allows for a one-to-one comparison with our full model involving both $\mathcal {P}$ and $\mathcal {R}$. To fit an unseen identity during testing, we initialize the $\textbf{z}_\text {env}$ with the environment map estimated from SIPR (Sun et al., 2020) trained on our lightstage dataset, followed by our two-step optimization process to reconstruct the unseen subject.

Our quantitative evaluations in Table 2 demonstrate that incorporating a dedicated $\mathcal {R}$ for relighting improves the overall performance significantly. As shown in Fig. 12, using only $\mathcal {P}$ fails to capture the environment illumination conditions completely. In contrast, relighting using OLATs obtained from $\mathcal {R}$ closely matches the ground truth lighting condition, thereby validating our design choice.

### Latent Space Design

The process of disentangling identity ($\varvec{\textbf{z}}_{id}$) and environment illumination ($\varvec{\textbf{z}}_{env}$) is executed in a data-driven approach. Leveraging our OLAT lightstage, we generate a range of lighting scenarios by combining these OLAT images with HDR environment maps. This allows us to synthesize natural illumination conditions for the subjects of the lightstage.

For each subject, we create 600 unique illumination scenarios by randomly choosing from a set of 2000 indoor (Gardner et al., 2017) and outdoor (Hold-Geoffroy et al., 2019) environment maps and combining them with the subject’s OLAT images. This gives us a collection of images depicting a single person under various illuminations, which we encode using a combination of  $\varvec{\textbf{z}}_{id}$ and 600 different  $\varvec{\textbf{z}}_{env}$ values. This principle is then extended to all the training subjects, each illuminated under 600 random lighting conditions.

It’s worth noting that within these 600 random illuminations, several lighting conditions are repeated across multiple subjects. As a result, we have multiple subjects sharing the same  $\varvec{\textbf{z}}_{env}$. When we train the $\mathcal {P}$ as an auto-decoder, we sample unique identity and illumination latent codes. This enables us to learn a disentangled representation of identity and illumination, with subjects under the same illumination sharing the same  $\varvec{\textbf{z}}_{env}$.

The primary benefit of this disentanglement is that it allows the extension of NeRF to handle a multitude of subjects and illuminations by utilizing latent conditioning. More specifically, the $\mathcal {P}$ can discern and accurately model details specific to both illumination and identity, such as face geometry. On the other hand, the $\mathcal {R}$ is solely responsible for modeling the face’s reflectance properties through One-Light-at-A-Time (OLAT) images. It’s well-established in computer graphics literature that precise modeling of reflectance requires a comprehensive understanding of geometry. While we do not explicitly condition the Reflectance Network with  $\varvec{\textbf{z}}_{i}$, we hypothesize that a disentangled latent space $\mathcal {P}$ provides the necessary accurate facial geometry features for the effective modeling of face reflectance.

**Fig. 13:**
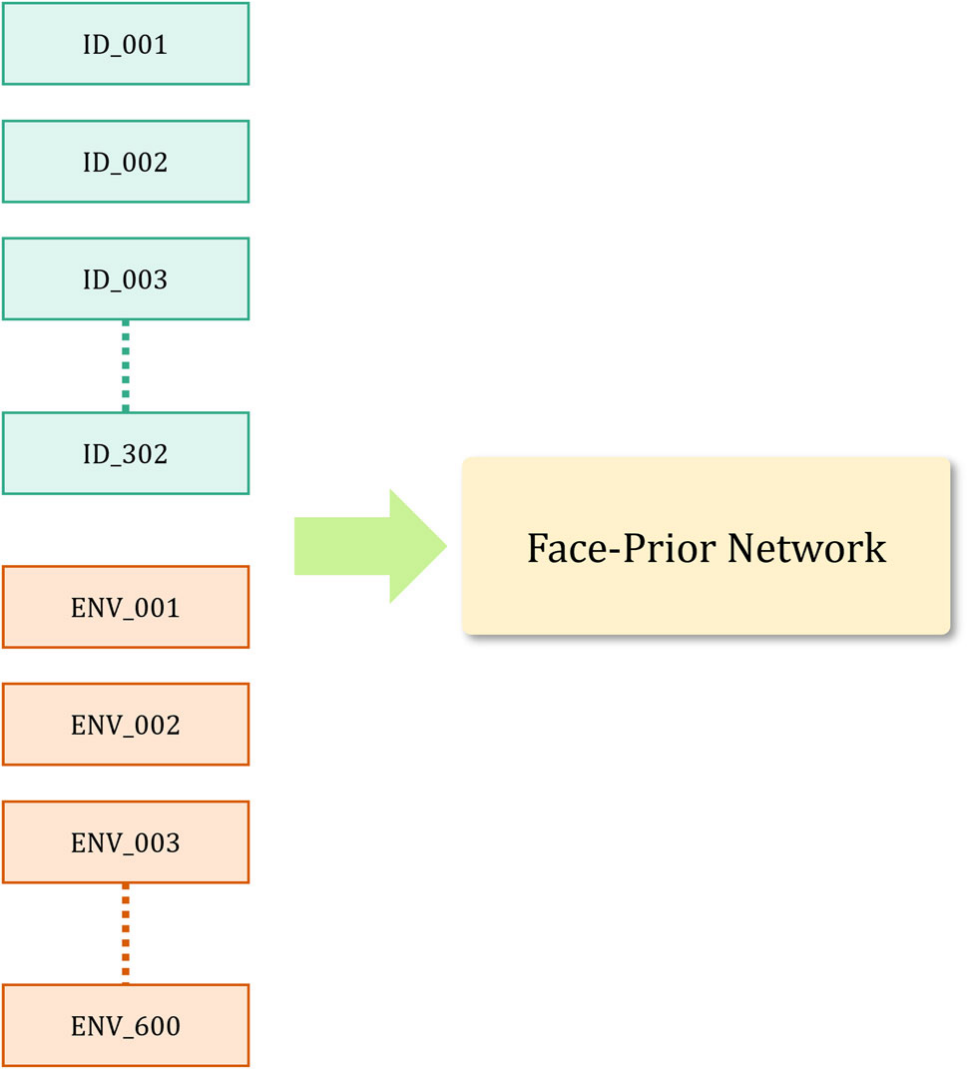
In the disentangled latent design, we store one $\varvec{\textbf{z}}_{id}$ per subject and one $\varvec{\textbf{z}}_{env}$ per illumination condition, amounting to 902 unique latent code

**Fig. 14:**
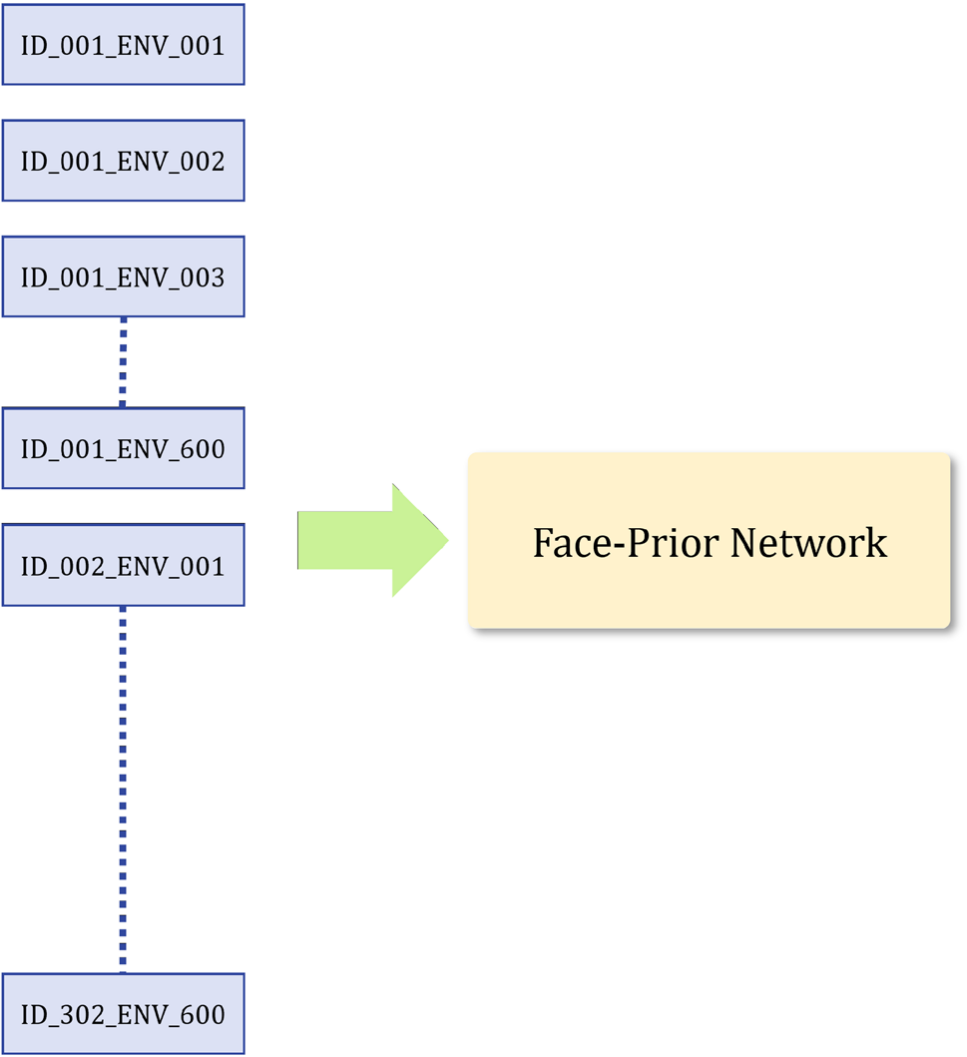
We relight each subject with 600 random environment maps. Thus naively mapping a single code for every combination of identity and lighting would lead to 181,200 unique latent codes

Another benefit of this disentanglement includes an efficient, shared latent space representation. Our approach uses separate latent codes for identity and illumination. During training, we store one $\varvec{\textbf{z}}_{id}$ per subject and one $\varvec{\textbf{z}}_{env}$ per illumination condition, amounting to 902 ($302~ \varvec{\textbf{z}}_{id}~+~600~\varvec{\textbf{z}}_{env})$ unique codes as shown in Fig. 13. Each identity code receives supervision under different lighting conditions. Similarly, each illumination code receives supervision from various subjects.

In contrast, if a single code was used for each *combination* of identity and lighting condition, we would need to supervise 181,200 ( $302~\varvec{\textbf{z}}_{id}~\times ~600~\varvec{\textbf{z}}_{env}$) unique latent codes. Codes representing the same subject under different illuminations would not be supervised jointly any more. To investigate this, we compare a “disentangled” model (i.e. 900 latent vectors) to one that uses one code per combination (i.e. 181,200 latent vectors). After training both models for an equal number of iterations, we tabulate our findings in Table 2: Having a single latent code for each identity and illumination leads to a combinatorial explosion of latent parameters, making it difficult to learn a good face prior. Figure 11 shows that using separate latent codes leads to better reconstructions of unseen subjects.

**Table 3:** Influence of latent space size

View synthesis		
	PSNR	SSIM
z = 16	22.73	0.57
z = 128	25.19	0.69
z = 256	25.44	0.80
z = 512	**26**.**67**	**0**.**82**

The table showcases the effect of varying the dimensionality of the latent space on the quality of novel view synthesis with three input views: Smaller latent space sizes are inadequate to represent both the identity and illumination information during testing. We find $z = 512$ to be optimal (indicated in bold)

## Parameter Study

  In this section, we discuss important parameters that influence our proposed method.

### Latent Space Dimensionality

**Fig. 15:**
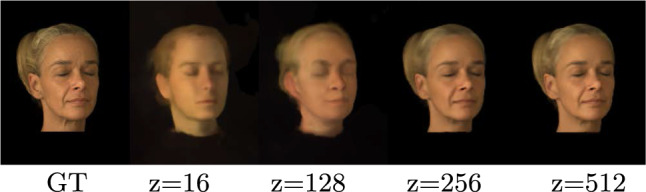
Impact of latent space size on novel view synthesis with three input views. The results indicate that small latent space sizes are inadequate for representing the identity and illumination information during testing

Our analysis, detailed in Sect. 7.4, underscores that our latent space representation, denoted as $\textbf{z}_j$, adeptly captures disentangled identity and illumination information. However, we discovered that this encoding demands a specific number of latent space dimensions. After conducting a series of qualitative and quantitative experiments, we established that a latent dimensionality of 512 provides optimal results, as presented in Table 3 and Fig. 15. Larger dimensionality for $\textbf{z}_j$ primarily inflates memory demands, while smaller ones prove to be insufficient in faithfully modeling both identity and illumination aspects. Therefore, balancing between memory considerations and the quality of results, we have set the optimal dimensionality of $\textbf{z}_j$ to 512. This space is equally apportioned between identity and illumination components, with dimensions allocated as 256 for $\textbf{z}^\text {id}_j$ and the remaining 256 for $\textbf{z}^\text {env}_j$.

### Reflectance Network Depth

The reflectance field of faces is modeled through OLATs by the *Reflectance Network*. Therefore, the network must have sufficient capacity to predict OLATs for any input $\omega _i$. We therefore investigate the impact of the depth of the reflectance network, evaluating networks with depths of 2, 4, and 8. Our results, summarized in Table 5, show that shallow networks (2 and 4 layers) are inadequate for learning a high-quality OLAT representation, as evidenced by lower PSNR and SSIM values. This is further demonstrated through qualitative results, presented in Fig. 17.

### Number of Training Identities

**Fig. 16:**
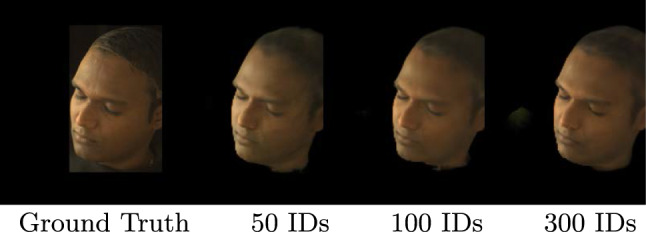
Even when we train our method on only 50 light stage identities, it produces good quality novel views on this unseen test subject

The *Face Prior Network* learns a distribution of faces captured under natural illuminations. In order to generalize to unseen identities, the network must be trained on a diverse set of identities. To determine the minimum number of training samples required for effective generalization, we trained multiple *Face Prior Network* models with 50 and 100 lightstage subjects and compared them to our finalized model, which was trained with 300 lightstage subjects. Surprisingly, we found that our method achieved comparable performance with as few as 50 training subjects, as demonstrated in Table 4. Even qualitative results showed very little variation between different models, as shown in Fig. 16.

**Table 4:** We summarize the impact on generalization with training identities

View Synthesis		
	PSNR	SSIM
50 IDs	25.42	0.81
100 IDs	26.34	0.81
300 IDs	**26**.**67**	**0**.**82**

We observe that as few as 50 subjects are sufficient to generalize to test subjects.

Best results are obtained with 300 training subjects (see bold)

**Table 5:** Reducing the depth of *Reflectance Network* hurts the scores for simultaneous relighting and novel view synthesis

View Synthesis + Relighting		
	PSNR	SSIM
$\mathcal {R}$ depth = 2	22.27	0.70
$\mathcal {R}$ depth = 4	22.58	0.74
$\mathcal {R}$ depth = 8	**22**.**79**	**0**.**76**

Best results obtained with depth = 8 (shown in bold)

**Fig. 17:**
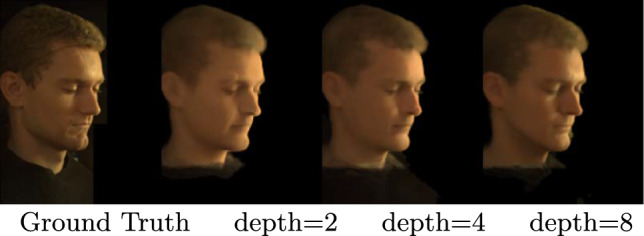
Reducing the depth of our *Reflectance Network* leads loss of fine-scale details and visible artifacts in the geometry (see right eyebrow)

**Table 6:** Influence of number of OLATs for the task of simultaneous relighting and view-synthesis

View Synthesis + Relighting		
	PSNR	SSIM
50 OLATs	19.70	0.72
100 OLATs	21.22	0.73
150 OLATs	**22**.**80**	**0**.**76**

Using all 150 OLATs gives the best results. In general, we observe that the quality of relighting improves with the increasing number of OLATs

**Fig. 18:**
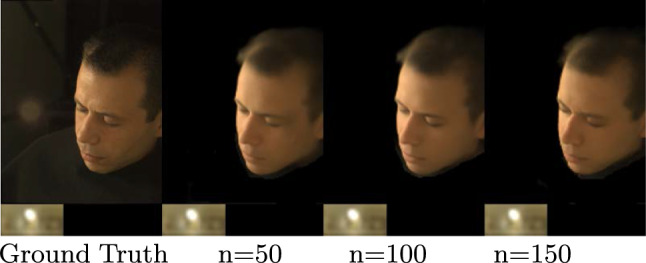
We show the significance of the number of OLATs (n) on final relighting. During simultaneous view synthesis and relighting, we observe that with fewer OLATs, the *Reflectance Network* struggles to accurately relight the environment illumination. Hence, using all the 150 OLATs of the lightstage dataset gives the closest resemblance to the ground truth

**Fig. 19:**
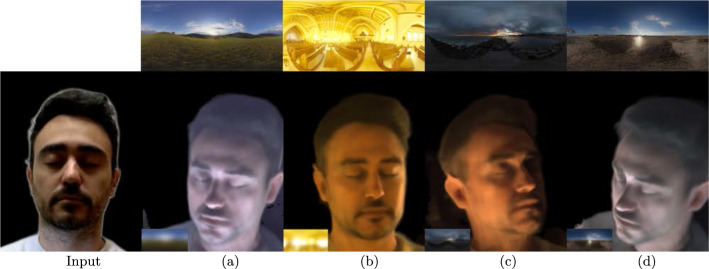
Relighting with text prompts. The top row shows the environment maps predicted by Text2Light. We use these maps to relight an unseen subject with a single input view. The text prompts used are **a** *A quiet, peaceful countryside with rolling hills and a bright blue sky.* **b** *Inside a church with yellow windows.* **c** *A rocky coastline with crashing waves and a lighthouse in the distance.* **d** *A serene, tranquil beach with soft sand and crystal-clear water*

### Significance of Number of OLATs

In this section, we examine the significance of the quality of relighting by utilizing different numbers of OLAT configurations: 50, 100, and 150 OLATs. We conduct evaluations for simultaneous view synthesis and relighting.

Since the original lightstage dataset contains 150 OLATs, we uniformly sample from the original configuration to select 50 and 100 OLAT configurations. Next, we train three different *Reflectance Network* models with various OLAT configurations for the same number of iterations. We summarize quantitative evaluations in Table 6 and observe that the quality of relighting increases with the increase in the number OLATs. This is distinctively clear from the Fig. 18, as the *Reflectance Network*  trained with 150 OLATs shows better results in comparison. We reason that an increase in the number of OLATs leads to a better approximation of the environment illumination and as a consequence, it improves the quality of relighting. In summary, we conclude that a higher number of OLATs improves the quality of relighting. In this work, we are restricted to 150 OLATs since it is the capacity of the lightstage dataset available to us.

## Application

This section presents an application for relighting using text-based prompts. We utilize Text2Light (Chen et al., 2022) to generate HDR environment maps based on textual input. To produce relit images, we combine the downsampled environment maps with the OLATs predicted by our method. Figure 19 displays some relighting results achieved with this approach.

**Fig. 20:**
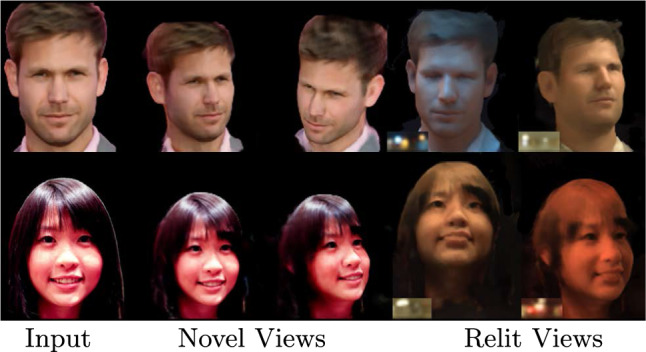
Given single input view from CelebA (top) and FFHQ (bottom). Although our method works well for novel view synthesis, it struggles to synthesize eyes and facial expressions during relighting

**Fig. 21:**
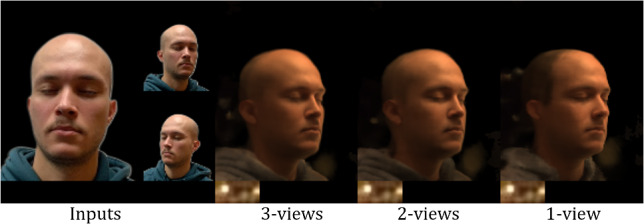
Our method produces good relighting and view synthesis using from 3, 2, or even 1 input view

## Limitations

Our proposed method generates high-quality photorealistic renderings, but it still has some limitations. In particular, we present the results of our approach on the FFHQ (Karras et al., 2021) and CelebA (Liu et al., 2015) datasets in Fig. 20. Although our model was trained on the lightstage dataset with subjects exhibiting closed eyes and neutral expressions, it can handle novel view synthesis with open eyes and natural expressions due to the fine-tuning of the *Face Prior Network* during testing. We show in Fig. 20 that our method preserves the mouth and eye shape during relighting, but it cannot synthesize their colors or texture. We argue that this is not a limitation of our approach but of the lightstage dataset. Lastly, under a monocular setting, our approach can sometimes generate regions that do not exist in reality. For instance, in Fig. 21 in the case of single input, hair is synthesized for the bald person. Such performance is expected due to insufficient information from a single view.

## Conclusion

We have presented an approach for editing light and viewpoint of human heads even with a single image as input. Based on neural radiance fields (Mildenhall et al., 2020), our method represents human heads as a continuous volumetric field with disentangled latent spaces for identity and illumination. Our method is designed to first learn a face prior model in an auto-decoder manner over a diverse class of heads. Further, followed by training a reflectance MLP that predicts One-Light- at-A-Time (OLAT) images at every point in 3*D*, parameterized by point light direction which can be combined to produce a target lighting. Quantitative and qualitative evaluations show that our results are photorealistic, view-consistent, and outperform existing state-of-the-art works.

## Figures and Tables

**Fig. 22:**
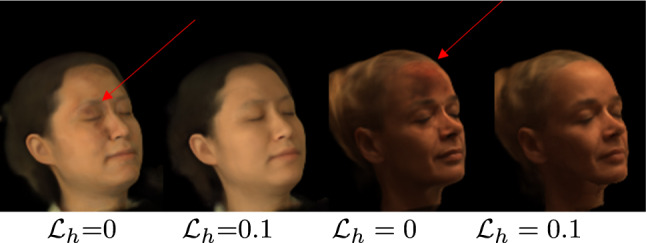
We visualize the importance of the hard loss ${\mathcal {L}}_h$ on the final results. Here, we show results from the view synthesis task. We use a default value of 0.1 for the hard loss. Removing the hard loss (${\mathcal {L}}_h=0$) produces significant cloudy artifacts as shown by the red arrows. Adding the hard loss (${\mathcal {L}}_h=0.1$) forces the volume to be more constrained around the head and thus removes such cloudy artifacts

**Fig. 23:**
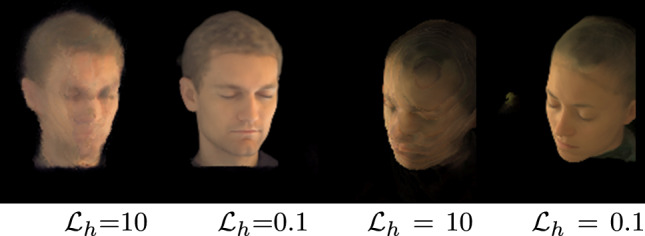
We visualize the impact of different values for the hard loss ${\mathcal {L}}_h$. The default value of the hard loss used in our experiments is 0.1. This figure shows that using an over-emphasized value of 10 leads to strong artifacts

**Fig. 24:**
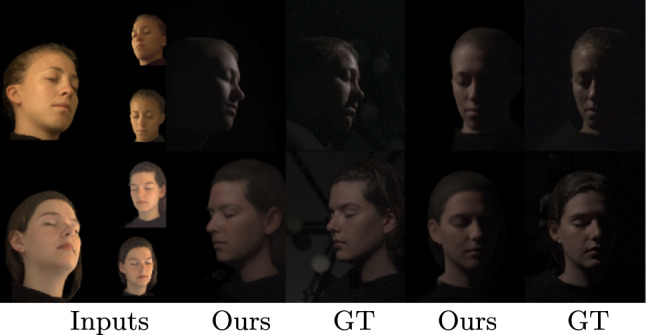
Using the *Reflectance Network*, we can synthesize OLAT images for an unseen identity. Our method captures view-dependent effects as well as accurate shadows and the result closely matches the ground truth

**Fig. 25:**
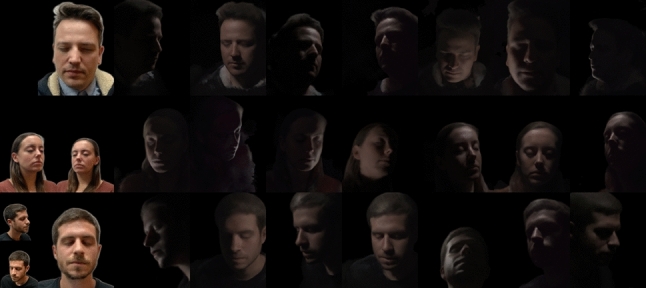
OLAT predictions of our method for the test subjects from the H3DS dataset. We show results with a single view as input (top), two views as input (middle) and three views as input (bottom). We render the predictions from different viewpoints. The OLAT predictions capture important illumination effects such as specularities and hard shadows

## Data Availability

Due to privacy concerns, we cannot make the dataset used in our project publicly available. However, to demonstrate the effectiveness of our proposed method, we evaluate our approach using publicly available datasets such as H3DS (Ramon et al., 2021): https://github.com/CrisalixSA/h3ds, FFHQ (Karras et al., 2021): https://github.com/NVlabs/ffhq-dataset, and CelebA (Liu et al., 2015): https://mmlab.ie.cuhk.edu.hk/projects/CelebA.html. Using these datasets allows us to evaluate the generalization ability of our proposed method on unseen data. H3DS provides high-quality 3D scans of human faces, FFHQ contains high-resolution facial images, and CelebA is a large-scale dataset of celebrity faces. We use these datasets to evaluate the performance of our proposed method in various scenarios, such as face rotation and relighting, and compare them with state-of-the-art methods.

## Appendix A

### A.1 Image-Based Relighting

The process of combining One-Light-at-A-Time (OLAT) images with High Dynamic Range (HDR) environment maps to simulate various illumination conditions is a well-established technique in the field of computer graphics, with roots dating back to the early 2000 (Debevec et al., 2000). This method provides a straightforward and effective way to generate realistic lighting effects and is particularly useful for rendering 3D models in various lighting conditions.

OLAT images are photos of the subject taken with lighting coming from a single direction at a time. When these images are taken from multiple directions, they form a detailed lighting profile of the subject from all angles.

HDR environment maps, on the other hand, represent a panoramic image of an environment that encodes the brightness of the light coming from every direction. Every pixel in these maps is equivalent to a light source and hence we model light coming from all directions of a sphere. These maps can capture the nuances of complex lighting conditions, including everything from the color of ambient light to the intensity of direct sunlight.

To combine an OLAT image with a target environment map, we first align the lighting direction in the OLAT image with the corresponding direction in the environment map. We then use the color and intensity values from the environment map to adjust the lighting in the OLAT image, effectively "relighting" the subject as if it were in the environment depicted by the map.

By repeating this process for OLAT images taken from multiple lighting directions and combining the results, we can create a single image of the subject as it would appear under the lighting conditions represented by the environment map. This technique enables realistic, data-driven lighting simulation from any viewpoint, which is essential for our work in portrait viewpoint and illumination editing.

### A.2 Multiple Light Sources

Real-world scenarios frequently involve multiple light sources. To reflect this complexity, our One-Light-at-A-Time (OLAT) based relighting method is designed to accommodate multiple light sources. The key advantage here is that our method neither requires explicit knowledge of the number of light sources, nor their precise positions, making it robust and flexible for diverse lighting conditions

In our proposed method, during the training phase, we re-illuminate our subjects using both indoor and outdoor environment maps, which naturally involve multiple light sources. This capability extends to our testing setting as well, and our results, especially those using FFHQ and CelebA datasets, demonstrate our model’s effectiveness under conditions of multiple unknown light sources.

However, when it comes to modeling face reflectance, we follow the principle laid out by Debevec et al. (2000), which suggests that to accurately model face reflectance, it is essential to illuminate the face with a single light source. This allows us to generate a reflectance map based on the direction of incoming light, which forms the basis of our Reflectance Network design.

Introducing multiple light sources at this stage would violate the necessary conditions for modeling reflectance, thereby disrupting the design principle. Nonetheless, this limitation does not prevent our method from effectively dealing with multiple light sources in real-world settings. We can re-illuminate the face to mimic the effects of multiple light sources by combining the predicted OLAT images with suitable environment maps. Therefore, our approach remains practical and applicable under conditions of multiple light sources.

## Appendix B

### B.1 Reducing Cloudy Artifacts

The background or boundary artifacts observed in the experimental figures are attributed to floating artifacts present within the NeRF volume. These artifacts are common in NeRF, especially due to inaccuracies in density values. During the training process of NeRF, color prediction is conducted by aggregating the sample points densities. Sometimes, regions that should be empty end up with non-zero density values, leading to inaccurate color values. These inaccuracies become particularly evident when we render images from novel viewpoints, as illustrated in our experimental figures.

**Table 7:** Impact of the hard loss ${\mathcal {L}}_h$ on novel view synthesis on the lightstage test set

	PSNR	SSIM
${\mathcal {L}}_h = 0.1$	**26.67**	**0.82**
${\mathcal {L}}_h = 0$	25.65	0.78
${\mathcal {L}}_h = 10$	19.81	0.64

Our default value of 0.1 for the hard loss produces the best results (indicated in bold)

We address this issue by striving to improve the density distribution through the use of ${\mathcal {L}}_h$. We further investigate the importance of the hard loss ${\mathcal {L}}_h$. This loss constraints *accumulation weights* $w_{r,k}$ to be sparse (Rebain et al., 2022), thereby encouraging the face geometry to approximate a surface. This measure prevents cloudy artifacts around the face as shown in Fig. 22 (see red arrows). In our main experiments, we use a default value of 0.1 for the hard loss. Figure 23 shows that using an over-emphasized value of 10 for the hard loss leads to severe artifacts. In Table 7 we examine the importance of the hard loss using quantitative evaluations against groundtruth. Here, we evaluate the lightstage test set. As expected, completely removing the hard loss leads to a strong drop in the PSNR and SSIM, as opposed to using it with the default value of 0.1. However, using an over-emphasized value of 10 leads to very poor performance. Finally, we want to point out that even though we significantly reduce the cloudy artifacts on the face region, sparse inputs make it hard to completely get rid of cloudy artifacts as seen in  Fig. 5. We believe this could be an interesting direction to explore in the future.

## Appendix C

### C.1 OLAT Predicitons

Figures 24 and 25 demonstrates One-Light-At-A-Time (OLAT) images produced by our method on the unseen subjects from the lightstage and H3DS datasets respectively. We show results using different numbers of input views and render the OLATs from different viewpoints. The predicted OLATs capture important illumination effects and details such as hard shadows and specularities.

## Footnotes

1 Code: https://github.com/prraoo/VoRF/.
